# Genetic repertoires of anaerobic microbiomes driving generation of biogas

**DOI:** 10.1186/s13068-018-1258-x

**Published:** 2018-09-20

**Authors:** Anja Grohmann, Yevhen Vainshtein, Ellen Euchner, Christian Grumaz, Dieter Bryniok, Ralf Rabus, Kai Sohn

**Affiliations:** 1 University of Stuttgart IGVP, Pfaffenwaldring 31, 70569 Stuttgart, Germany; 2 Fraunhofer IGB, Nobelstrasse 12, 70569 Stuttgart, Germany; 3 University of Applied Science Hamm-Lippstadt, Marker Allee 76–78, 59063 Hamm, Germany; 4 Institute for Chemistry and Biology of the Marine Environment (ICBM), University of Oldenburg, Carl-von-Ossietzky-Strasse 9-11, 26111 Oldenburg, Germany

**Keywords:** Hybrid assembly, Biogas plant, Microbiome, Metatranscriptome, BioMETHA, EC reference sequence collection, Methanogenesis, Metagenome

## Abstract

**Background:**

Biogas production is an attractive technology for a sustainable generation of renewable energy. Although the microbial community is fundamental for such production, the process control is still limited to technological and chemical parameters. Currently, most of the efforts on microbial management system (MiMaS) are focused on process-specific marker species and community dynamics, but a practical implementation is in its infancy. The high number of unknown and uncharacterized microorganisms in general is one of the reasons hindering further advancements.

**Results:**

A Biogas Metagenomics Hybrid Assembly (BioMETHA) database, derived from microbiomes of biogas plants, was generated using a dedicated assembly strategy for different metagenomic datasets. Long reads from nanopore sequencing (MinION) were combined with short, more accurate second-generation sequencing reads (Illumina). The hybrid assembly resulted in 231 genomic bins each representing a taxonomic unit with an average completeness of 47%. Functional annotation identified 13,190 non-redundant genes covering roughly 207 k coding sequences. Mapping rates of metagenomics DNA derived from diverse biogas plants and laboratory reactors increased up to 73%. In addition, an EC (enzyme commission) reference sequence collection (ERSC) was generated whose genes are crucial for biogas-related processes, consisting of 235 unique EC numbers organized in 52 metabolic modules. Mapping rates of metatranscriptomic data to this ERSC revealed coverages of up to 93%. Process parameters and imbalances of laboratory reactors could be reconstructed by evaluating abundance of biogas-specific metabolic modules using metatranscriptomic data derived from various fermenter systems.

**Conclusion:**

This newly established metagenomic hybrid assembly in combination with an EC reference sequence collection might help to shed light on the microbial dark matter of biogas plants by contributing to the development of a reference for biogas plant microbiome-specific gene sequences. Considering a biogas microbiome as a complex meta-organism expressing a meta-transcriptome, the approach established here could lay the foundation for a function-based microbial management system.

**Electronic supplementary material:**

The online version of this article (10.1186/s13068-018-1258-x) contains supplementary material, which is available to authorized users.

## Background

Anaerobic digestion (AD) combines organic waste management with the generation of biogas (methane), as a renewable source of energy. Biogas is easily storable and continuously available in contrast to fluctuating energy from sun and wind. Due to progress in the field of energy transition, the need for such a balanced energy source will increase in the future with a promising growth potential [1].

Although microbial communities are fundamental for biogas production, the regulation of the underlying processes is still based on optimization of technical and chemical parameters yet. The requirements of the involved microbial communities are taken into consideration by, e.g., adjusting the C:N ratio or supplying nutrients and trace elements. In contrast, specific requirements of microorganisms essential for the process are not considered yet. However, the need for microbial management and monitoring systems (MiMaS) has been highlighted and many efforts have been made to optimize them [2, 3]. Accordingly, there are strategies that focus on a better characterization of the respective microorganisms, whereas others try to define process-specific marker species and related community dynamics [2]. Unfortunately, fundamental knowledge on anaerobic biogas microbiomes to establish a reliable MiMaS is still lacking to facilitate a robust and flexible operation of biogas plants [2]. Consequently, existing databases still are limited to fully cover the phylogenic diversity and metabolic potential of many different environmental sources used to seed biogas plants.

Next-generation sequencing (NGS) has boosted our understanding of complex, poorly characterized biological systems. This technology generates metagenomic as well as metatranscriptomic data, providing blueprints for the composition and functional diversity of microbial communities of environmental samples [4]. Furthermore, it allows identification of unknown, non-cultivable microbial organisms and insight into functional processes of specific ecosystems. In particular, whole genome shotgun (WGS) sequencing represents a powerful approach which allows de novo assemblies for a more accurate assignment of species to distinct taxa and also serves as a basis for in silico gene annotation to reveal functional properties. A biogas-related database containing 236 nearly complete genomes, each representing a *bona fide* species, has been published recently [5, 6]. By this database as a reference to map metagenomic DNA from biogas microbiome samples, it became possible to provide a coverage rate of nearly 50% and to suggest marker species relevant for process recovery for the first time [7]. However, knowledge about the functional role of identified species is still limited, as a functional annotation for this database is not yet published, further hindering a deeper understanding of the correlation between genomic potential and expressed functions of microbial communities relevant for biogas production. Consequently, functional annotation of de novo assembled metagenomes of biogas microbiomes will shed light not only onto microbial dark matter, but also on their functional role.

The aim of this work was to expand on biogas microbiome-specific databases, to more comprehensively cover respective metagenomes and to facilitate functional evaluation of biogas processes. Within this work, a hybrid assembly workflow was adapted for the usage of metagenomic data combining long reads from nanopore sequencing (MinION) with short, more accurate NGS reads (Illumina) resulting in a Biogas METagenome Hybrid Assembly (BioMETHA-) database with a comprehensive functional annotation. Thus, BioMETHA should allow an enhanced characterization of the relevant microbial community, not only with regard to their taxonomic diversity and functional potential, but also on the level of activated pathways relevant for biogas production. In combination, with a manually curated EC reference sequence collection (ERSC), covering known biogas relevant pathways, it provides targeted evaluation of transcriptomic data and the functional potential of biogas plants. Our findings might support the concept of a biogas microbiome as a single complex system comprising expression of relevant metabolic modules also driving cross-species interactions.

## Results and discussion

### Establishment of BioMETHA—a comprehensive biogas metagenomics database

**Fig. 1:**
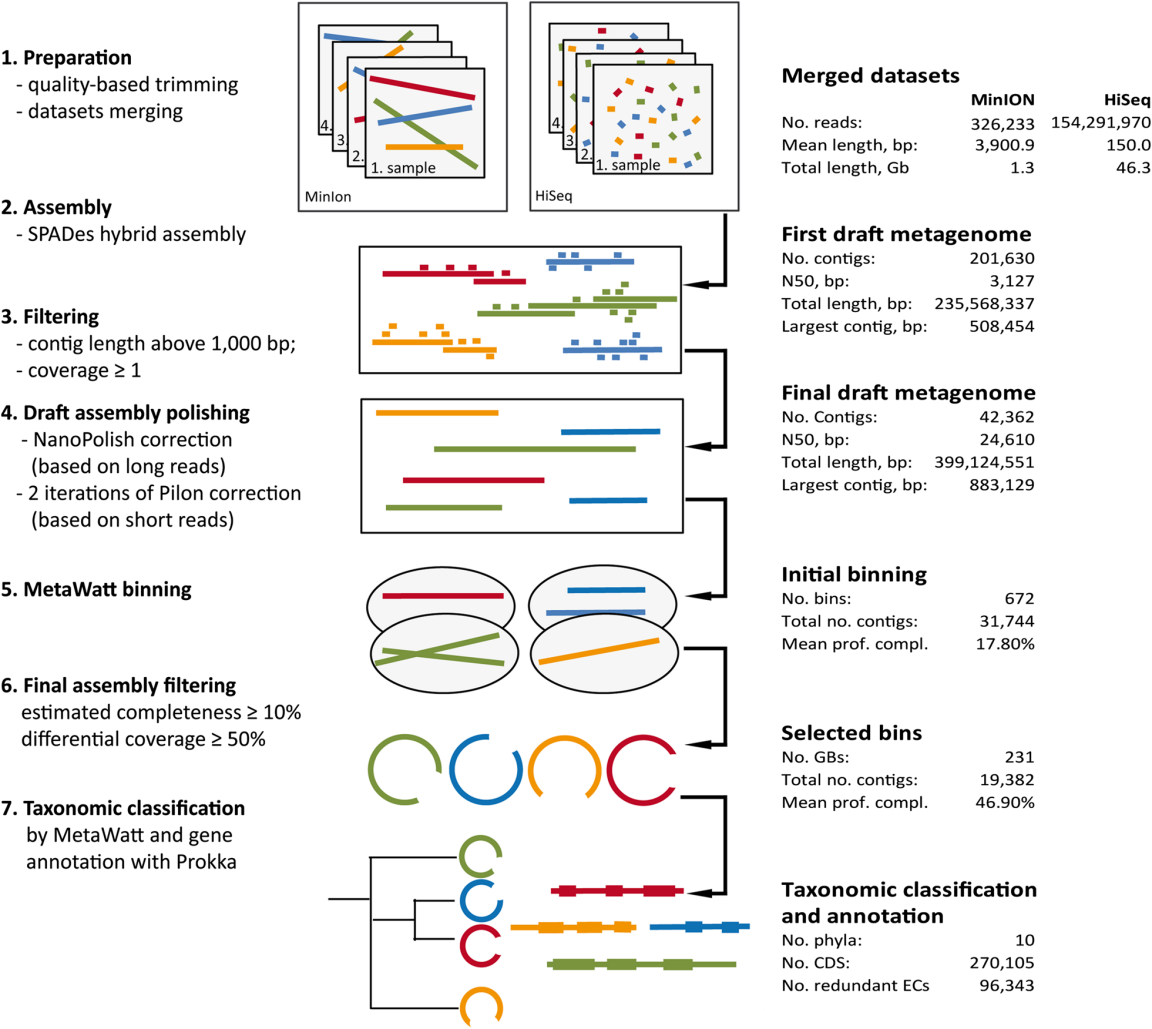
Metagenomic hybrid assembly workflow. On the left panel all processing steps are listed while on the right panel all intermediate workflow statistics are listed

Assembly of short sequencing reads has some inherent difficulties related to genomes containing tandem repeats that can span over thousands of bases [8]. Such regions cannot be assembled satisfactorily using solely second-generation high-throughput sequencing. In contrast, third-generation sequencing operates with long reads and, therefore, can resolve difficulties in assemblies much more reliably. Using a hybrid assembly strategy, combining both long and short reads will help to overcome difficulties for assembly and making computation more efficient. For BioMETHA assembly, we combined 154.3 million Illumina quality-trimmed read pairs (approximately 46 Gbs) with 326,223 quality-trimmed MinION reads revealing a mean length of 3852 bp generated from biogas plants 1–4 (Table 1 and Additional file 1). We pooled quality-trimmed reads from all samples to achieve higher average coverage and to increase overall reliability of the dataset [9]. As currently published hybrid algorithms mostly are just focused on assembly of individual genomes [10, 11], we adopted a pipeline based on the SPAdes hybrid assembler, for the assembly of our metagenomic sequences from biogas plants. The resulting first draft genome includes 201,630 contigs with a total sequence of 235,568,337 bp and a mean sequence length of 1168.32 bp (Fig. 1). To improve the assembly, two additional steps were implemented: correction of the draft assembly using NanoPolish, and 2 further iterations of the resulting draft metagenomes using Pilon. Accordingly, we reduced the number of contigs to 42,362, while N50 increased from 3127 to 24,610 bp with a mean sequence length of 9421 bp. This final draft metagenome represents a significant improvement over recently published approaches from other groups including a N50 of 12,418 bp from Güllert’s group [12], as well as for the Symbio database with a N50 of 17,256 bp [5, 6]. Subsequently, we used MetaWatt to assign 42,362 contigs from our final assembly into genomic bins (GBs). 672 GBs were generated comprising 357 million nucleotides in total and an average profile completeness of 17.8%. We removed 12,362 of the shortest contigs, roughly representing 18% of the total assembly length that did not map at least two out of four metagenomic samples generated with Illumina. Finally, 231 GBs were obtained with profile completeness of 10% or more, increasing the average profile completeness to 47% (Fig. 1) whereby genome completeness was calculated based on 137 marker genes selected by Campbell et al. [13], Among them, 98 GBs have an estimated profile completeness above 50% and 15 GBs above 90%. The final 231 GBs were then used for further taxonomic classification and gene annotation revealing 10 phyla (Additional file 2). 127 GBs affiliate with the *Firmicutes* phylum, with 88 among those belonging to the *Clostridiales* order and 40 to the *Clostridia* class; the latter harbors the families *Clostridiaceae*, *Ruminococcaceae* (3 GBs each) and *Lachnospiraceae* (1 GB). Twelve GBs belong to the second largest phylum of the data set, namely the *Bacteroidetes*, with 7 members from the *Bacteroidales* order and one from the *Petrimonas* genus. Such distribution of GBs from a biogas plant-derived metagenomic assembly is in agreement with previous findings [5, 6, 14, 15]. Furthermore, the ratio between GBs belonging to *Firmicutes* and *Bacteroidetes* phyla is in accordance to the data from other biogas fermenters [12, 16]. The BioMETHA database also includes the bacterial phyla *Chloroflexi* (5 GBs), *Synergistestes* (2 GBs), *Ternicutes* (2 GBs), *Fibrobacteres* (2 GBs) and *Thermotogae* (1 GB) which can be also found in the Symbio database [5, 6]. In addition, identified *Candidatus Cloacimonetes* phyla (3 GBs) and *Planctomycetes* (1 GB) were also already described to be associated with biogas plant microbiomes [17, 18]. Sixty bacterial GBs could not be unambiguously classified at the phylum level. Nine GBs belong to the *Euryarchaeota,* seven of which could be classified at least to the family level. Another seven GBs were marked as hybrid as they could not be clearly assigned either to bacteria or archaea possibly due to a very low estimated completeness of below 20%.

**Table 1:** Overview of the samples from agricultural biogas plants and laboratory reactors used in this study, showing the different operational parameters and the type of sequencing approaches applied

Fermenter	Operational parameters		Database assembly sequencing approach	Database evaluation sequencing approach	
	Substrate	Temperature, °C	DNA seq. HiSeq and MinIon sequencing	DNA seq. HiSeq sequencing	RNA seq. HiSeq sequencing
Agricultural biogas plant					
1	Maize silage, cattle slurry, horse + pig + cattle manure, organic waste	41	×		
2	Maize silage, pig + cattle slurry, chicken dung, organic waste	54	×		×
3	Maize silage, grass silage	52	×		×
4	Maize silage, grass silage	40	×		
5	Maize silage, cattle slurry, horse manure	51		×	
6	Maize silage	40		×	
7	Grass silage, maize silage, grain, cattle slurry	40		×	×
Laboratory reactors					
R1/R2 d0	Maize silage	41		×	×
R1 7 d	Maize silage	41		×	×
R1 21 d	Maize silage	41		×	×
R1 42 d	Maize silage	41		×	×
R1 84 d	Maize silage	41		×	×
R2 7d	Maize silage	35		×	×
R2 21d	Maize silage	41		×	×
R2 42 d	Maize silage	41		×	×
R2 84 d	Maize silage	41		×	×

Finally, coding sequences (CDS) were predicted and annotated for these 231 GBs resulting in 270,105 predicted CDS out of which 96,343 could be assigned to EC numbers. The assembly consists of 13,190 non-redundant transcriptional units, comprising 1873 unique EC numbers. To further complement the functional annotation, we additionally annotated every GB using InterProScan. It was able to match successfully about 1.97 millions of protein signatures from different databases such as KEGG, Pfam, PANTHER and could also assign 779,094 GO terms.

The BioMETHA database and the corresponding annotations are available for download from European Nucleotide Archive web page under the PRJEB27149 accession number.

### Analyses of biogas microbiomes at the metagenomic level

One of the aims of the assembly was to improve the mapping coverage for metagenomic data derived from biogas plant samples to enable a more comprehensive understanding of the biological processes underlying biogas production. To test our assembly, metagenomic sequences from production scale agricultural biogas plants 5 to 7—that were not used for assembly—were mapped to the annotated BioMETHA or to the Symbio database. On average, we could uniquely map 60% of the reads to the 231 GBs of the BioMETHA assembly, whereas 46% of the reads were mapped to the 236 GBs of the Symbio database. For samples derived from biogas plant 7, we increased the mapping rate by 17%. Subsequently, we also checked to which extent unmapped reads following mapping either to BioMETHA or to Symbio can be mapped to the respective other database. 24.3% of reads unmapped to BioMETHA could be further mapped to Symbio, while 41.5% of reads unmapped to Symbio could in turn be mapped to BioMETHA. Mapping results indicated that both databases contain unique sequences not present in the respective counterpart, implicating that reconciling both databases holds promise for even more comprehensive coverage in the future.

### Analyses of biogas microbiomes at the metatranscriptomic level

Using BioMETHA, we achieved a significant increase in mapping rate compared to currently available metagenome databases from biogas plants, facilitating the investigation of specific characteristics and roles of different genera and families within the respective microbial community. However, each metagenomic assembly that increases the phylogenetic diversity of known species still represents just the tip of an iceberg compared to the unknown microbial dark matter [19]. Therefore, especially for such complex and dynamic microbial communities like biogas plant microbiomes, it is challenging to reach 100% of sequence mapping on the species level. Hence, we extended our study on the applicability of expression analyses of the genetic repertoire which is established by the respective microbiome to better describe and understand metabolic processes in biogas plants. To get a general view on GO term representation that was implicated in biogas production, we mapped meta-transcriptomes from high producing reactors R1 and R2 (day 42 and 84) to BioMETHA and analyzed the top 500 expressed transcripts for GO terms. Among the 30 most prominent GO term categories, methanogenesis ranked third place even ranking higher than ribosome and translation (Additional file 3). Higher coverage only was found for “oxidation–reduction process” and “DNA-binding” holding promise for the identification of novel genes not yet described to be critical for biogas production (Additional file 4). Additionally, we also analyzed the meta-transcriptomes from samples of low and high level producing biogas reactors (different time points of R1/R2). Clustering of the top 500 most differentially expressed transcripts showed a clear distinction of low and high level biogas samples (Additional files 5, 6) indicating that this kind of expression signature might support optimization of biogas production processes in the future.

To provide a more targeted view on genes involved in biogas production, a comprehensive pathway map, summarizing all biological processes relevant to anaerobic digestion pathway and conversion of common carbon sources into methane, was manually reconstructed. The corresponding EC numbers, representing numerical classifiers for enzymes and enzyme-catalyzed reactions derived from different organisms already described by literature search to be implicated in biogas generation, were integrated and combined to metabolic modules summarized in a pathway map (Additional files 7, 8). In addition, we also included lipases (EC 3.1.1.-; EC 3.1.1.3; EC 3.1.1.23) and proteases (EC 3.4.-.-) to complete for hydrolysis processes (Additional file 9).

**Fig. 2:**
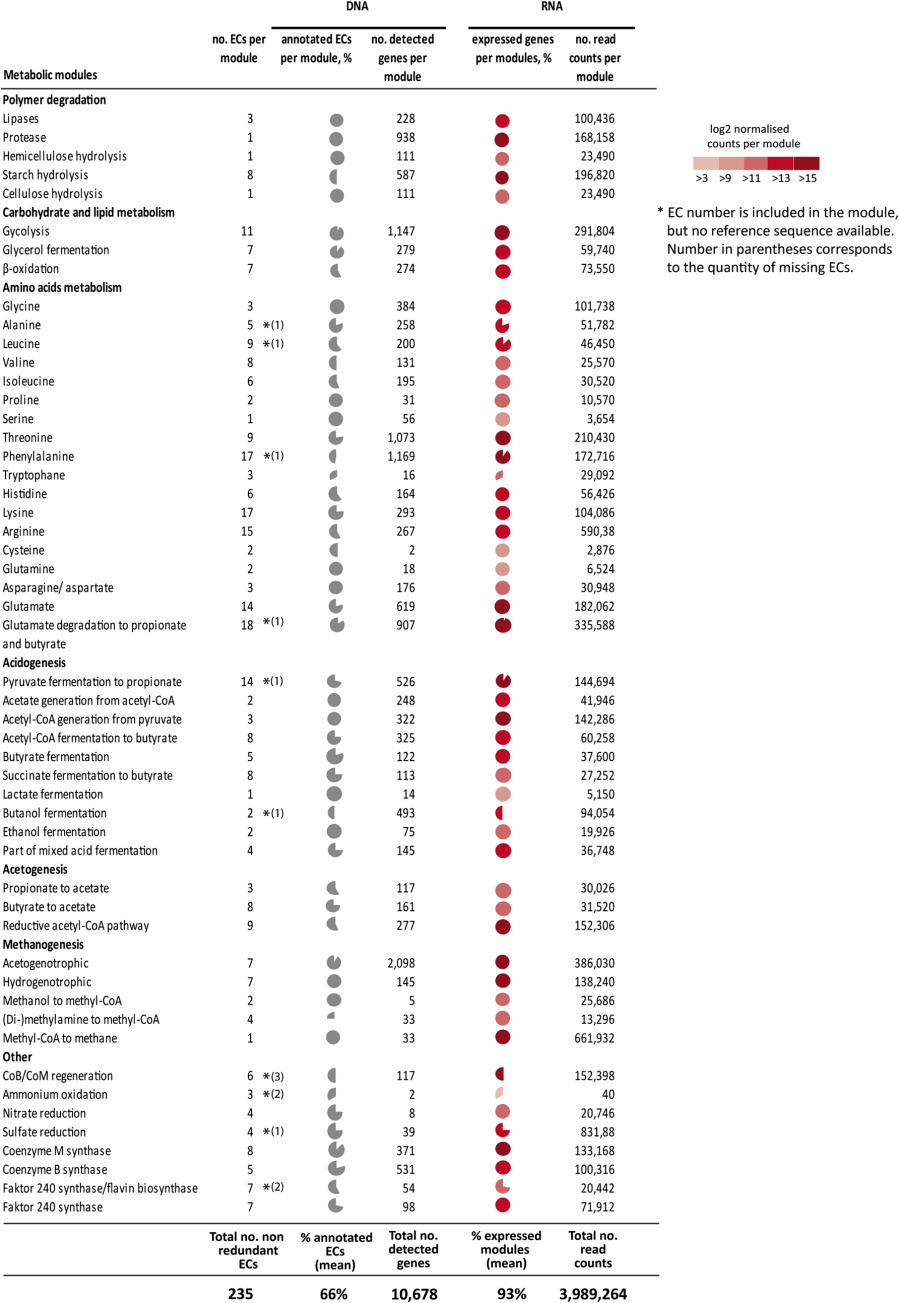
Biogas production pathway-related EC modules. Detailed information about AD pathway can be found on the overview map in Additional file 3: Figure S1. On the left all enzymatic reactions modules are listed, grouped by categories. Corresponding numbers of ECs in each module are in column 2 (number of asterisks indicating number of ECs without known sequence). Some modules may include redundant EC numbers. Gray pie-charts in column 3 representing the % of BioMETHA-annotated ECs per module while numbers in column 4 represent number of detected genes per module, based on DNASeq read counts. Colored pie-charts demonstrating % of genes expressed in each module with colors representing log2 transformed normalized RNASeq reads counts per module from biogas plant 2, 3 and 7

In total, 10,678 genes derived from BioMETHA could be assigned to the functional categories implicated in biogas generation, representing 156 (66%) out of the 235 biogas relevant EC numbers (Fig. 2). All metabolic modules are at least represented to 25% by BioMETHA. In particular, degradation of polymers and further carbohydrate and lipid metabolism are well represented by the newly annotated genes (~ 90%). Metabolic modules relevant to amino acid metabolism show higher variance, while glycine, proline, serine, glutamine and the asparagine/aspartate metabolic routes are completely covered. For tryptophan metabolism, only one out of three EC numbers could be annotated. The methanogenesis modules which are attributed to only 9 archaeal GBs are well covered, with the only exception of the (di-)methylamine to methyl-CoA pathway (25% coverage). One possible explanation to such variability in coverage among metabolic modules could be the fact that archaea are much better characterized in the microbiomes of biogas plants as compared to the more complex bacterial communities [5]. In general, methylamines can be used as substrate only by a small number of Archaea. Therefore, it is not unexpected that this pathway might be underrepresented in metagenomic assembly [20]. Several metabolic modules needed for coenzyme or ammonium oxidation are not fully covered either by BioMETHA (asterisks in Fig. 2) or publically available EC reference sequence databases, such as KEGG or RefSeq.

To perform expression analyses on the metatranscriptomic level, the gene sequences from publically available databases of all EC numbers implicated in biogas generation were combined with the corresponding gene sequences from BioMETHA (156 EC numbers) to establish a most updated EC reference sequence collection (ERSC). However, a few relevant EC numbers could not be annotated using BioMETHA. This might be due to inaccuracies of the binning strategy or due to underrepresentation of species in the microbial population and, therefore, lack of sequencing depth [21, 22].

To test the feasibility of the established ERSC, we performed RNA-seq experiments from samples of three agricultural biogas plants (Bgp 2, 3 and 7). The total number of reads was calculated for every metabolic module based on read counts assigned to individual EC numbers constituting the modules (Fig. 2). The abundance of individual metabolic modules was calculated as log2-transformed mean RPKM value for every EC gene sequence of the respective module. In contrast to the metagenomic data, metatranscriptomic data revealed a much higher coverage of respective EC categories (93% compared to 66%; Fig. 2). Out of 52 metabolic modules only, 12 could not be covered completely by the RNA-seq data. Overall, from 235 ECs, 219 could be found to be expressed in at least one of the investigated biogas plants. Consequently, nearly all enzyme sequences relevant for the microbiome of a biogas plant could be deduced using the ERSC in combination with our functional annotation following hybrid assembly. Therefore, at the transcriptomic level, a more complete functional picture of the underlying biogas processes could be provided than just by the classical taxonomic analyses of microbial communities at the species level.

**Fig. 3:**
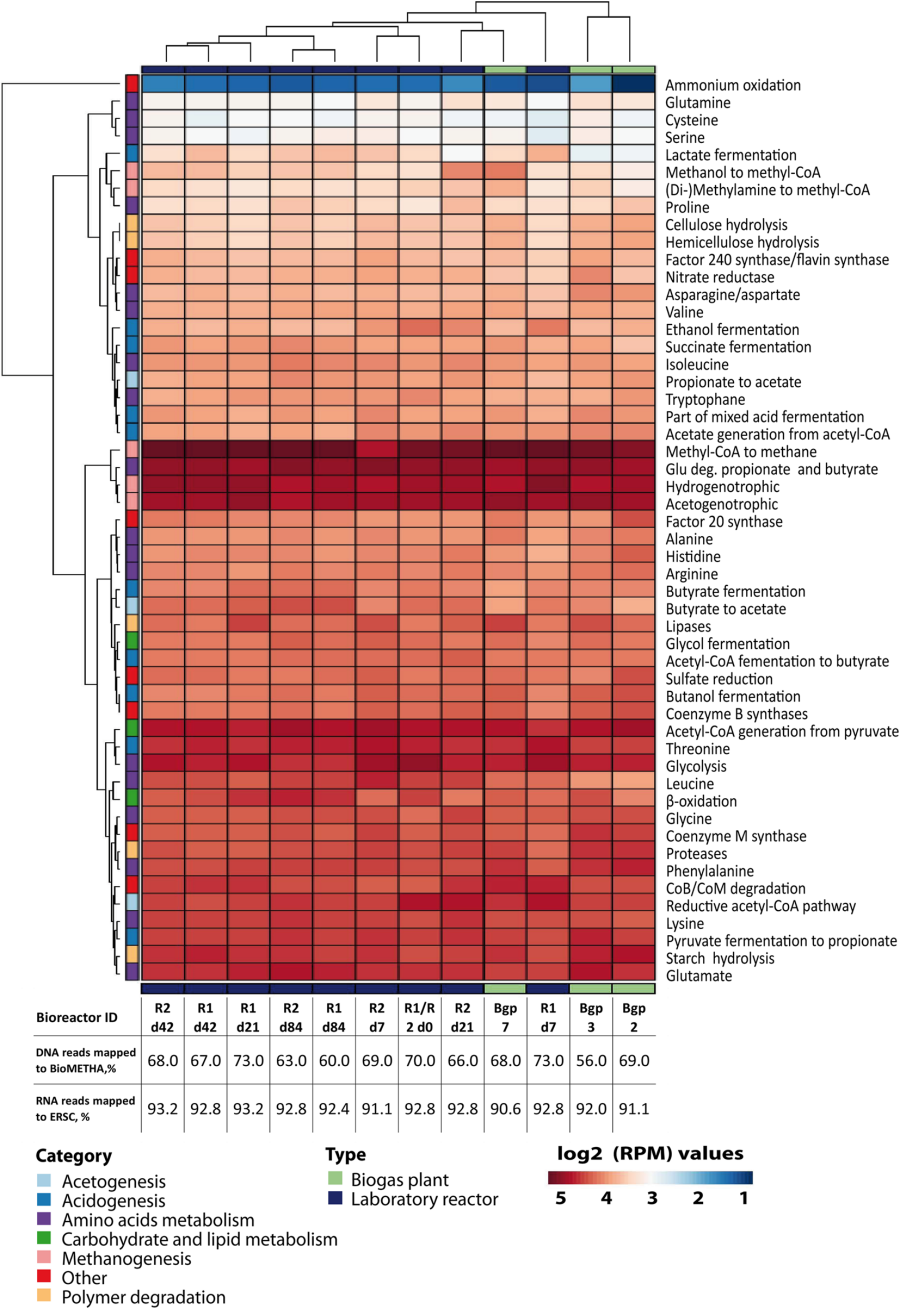
Comparative metatranscriptomic analysis of biogas plants. Heatmap colors representing log2 RPM values per module. Hierarchical clustering was done for samples as well as for modules. Corresponding dendrogram on the right shows clustering of biogas plants and laboratory reactors. The dendrogram for modules clustering is not shown. All modules correspond to modules listed in Fig. 2

In the next step, we wanted to figure out how metatranscriptomes per se can be used to differentiate between various microbiomes derived from different reactor types or from different time points during biogas fermentation. Consequently, we analyzed the metatranscriptomes of three agricultural biogas plants (Bgp2, Bgp3 and Bgp7), but also of two laboratory scale reactors fed only with maize silage (R1, R2) and assigned respective expression data (reads per million) towards the individual functional categories (Fig. 3). Additionally, the respective mapping rates of the individual samples to BioMETHA as well as the percentage of the 235 ECs covered by the metatranscriptomes are listed (Fig. 3). These data show that by mapping DNA sequence reads to BioMETHA, we could increase the mapping rate to 68% on average for the laboratory reactor samples compared to 47% when mapping to the Symbio database as previously reported [7]. Cluster analysis of samples revealed that the two thermophilic biogas plants Bgp2 and Bgp3 cluster together regardless of their substrate components. This result is in good agreement with the well-known effect of temperature on biogas plant performance and community structure [23–26]. Clustering also confirms time-dependent differences of lab reactors R1 and R2. While R2 experienced an unintended initial process disturbance with a temperature of 35 °C until day 16, R1 was running with a process temperature of 41 °C directly from the beginning. It has been shown that the composition of microbiomes and respective metatranscriptomes of the two fermenters conformed after day 21 [7]. Hence, samples from R1 and R2 from days 42 and 84 are clustering together, while they show clear deviations on days 7 and 21. Having a look at the expression of metabolic modules in more detail, it is obvious that the *mcr* expression (related to the conversion of methyl-CoA into methane (EC 2.8.4.1) was negatively affected by the process disturbance as well as the genes involved in hydrogenotrophic methanogenesis are expressed at lower levels in R2 at day 7 compared to R1 at the same and later time points in the process (Additional file 10 and Fig. 3).

The combined DNA and RNA data from microbiomes of all studied biogas production systems (Fig. 2) revealed the varying importance of the individual degradation modules. In case of polymer degradation, genes and transcripts for proteases and starch hydrolysis are conspicuously abundant agreeing with the composition of the feed material (Table 1). On the level of small metabolites, glutamate apparently plays an important role for catabolic and anabolic purposes alike. Finally, as expected, genes and transcripts involved in methanogenesis are particularly abundant. Notably, while the number of genes for hydrogenotrophic methanogenesis is comparably low in number, they display high expression levels.

To date, there have been lots of efforts to monitor the complex microbial communities, microbial population dynamics and the effects of process parameters and disturbances during biogas production. However, none of them was able to bring forward standard characteristics on the microbiome to implement microbial management into application [2]. In contrast, the relevant pathways to convert polymers into methane are less alterable and complex, than the underlying community structures, and seem to be, therefore, more promising for an implementation in process control. Nevertheless, a strateg, based on transcriptomic data will become even more reliable, if environmental-specific reference sequences are available [27]. Thus, the establishment of BioMETHA and its accompanying EC reference sequence collection might provide a useful instrument for a better understanding and controlling of biogas processes. In this context, repeatability of testing results is an important issue. Therefore, a prerequisite for a maximum of repeatability for metagenomics and meta-transcriptomics data from future studies is that the corresponding databases should be as comprehensive as possible. In this respect, our BioMETHA and the EC reference sequence collection represent the most comprehensive databases currently applicable. Nevertheless, additional efforts in the future have to be made to further improve such databases which includes, but is not limited to, the analyses of other types of biogas plants as well as the raising of sequencing depth to provide more comprehensive sequencing data to further improve assemblies and to complete genomic bins.

## Conclusions

This newly established metagenomic hybrid assembly in combination with an EC reference sequence collection might help to advance our functional understanding of microbiomes from biogas plants. In particular, this strategy complements the traditional biodiversity-centered approaches by a database of genes/transcripts directly involved in the anaerobic digestion and thereby in the process of biogas formation. From these databases, truly functional biomarkers might be derived for online monitoring and controlling of the process performance in biogas plants particularly those fed with agricultural wastes including silage and animal manures. The database can be extended and completed with the methods reported here, e.g., using samples from biogas plants utilizing other substrates.

Moreover, considering a complex microbiome as a meta-organism expressing a meta-transcriptome might be a promising approach for improving the knowledge about metabolic sequences in methanogenic microbial and developing a function-based microbial management system, which could be used in future to optimize biogas production.

## Methods

### Biogas plant characteristics and sample collection

For this study, samples were taken from seven different agricultural biogas plants with different substrates and process temperatures (Table 1). While biogas plants 1–5 are located in the federal state of Baden-Württemberg, biogas plants 6 and 7 are located in the federal state of Lower Saxony. Samples were collected from biogas plants in two 2-ml reaction tubes and additionally in two 50-ml falcon tubes and directly frozen on dry ice. For long-term storage, the samples were kept at − 20 °C until DNA/RNA extraction.

Additionally, samples from two anaerobic digestion bioreactors (R1 and R2) which were carried out in a continuous biogas test (CBT), were analyzed. In R2, a delay in process temperature control was initiated from the beginning of the fermentation leading to an initial acidification of the anaerobic sludge. A detailed description of the processes has been recently published [7].

### Nucleic acid extraction

DNA from biogas plants 1–7 was extracted using the ZR Fecal DNA MiniPrep™ Kit (Zymo Research; Irvine, USA). Cell lysis was performed with a high-speed cell disturber Precellys^®^ 24 Homogenisator (VWR, Germany) for 40 s at 5000 rpm. Additional cleanup of the isolated DNA was performed by Agencourt AMRure XP beads from Beckman Coulter (Brea, CA, USA). Isolated DNA was stored at − 20 °C until library preparation. RNA was isolated using the ZR Soil/Fecal RNA MicroPrep™ Kit (Zymo Research, Irvine, USA). Isolated RNA was stored at − 80 °C until further preparation steps. The qualities of nucleic acids were checked with the HS NGS and HS RNA Fragment Analysis Kit on a Fragment Analyzer (AATI, USA).

Nucleic acids from R1 and R2 samples were isolated according to a protocol published recently [7].

### Library preparation and quality control

For Illumina sequencing samples used for the assembly were prepared using the TruSeq DNA PCR-Free Library Preparation Kit (Illumina, San Diego, CA, USA). According to the manufacturer’s protocol, the fragmentation step of the genomic DNA was done using 3000 ng genomic DNA and 2 µl Reaction Buffer v2; total volume was adjusted to 18 µl with water. The reaction mix was incubated for 18 min at 37 °C. For size selection, we used the Bluepippin system with 2% DF Marker M1 Gel and a size range selection of 400–800 bp (Beverly, MA, USA). The following end repair step was finished by a cleaning step without additional size selection. Additional samples from biogas plants were not used for the assembly but for metagenomic analyses. Samples R1 and R2 were prepared following Illumina’s Nextera DNA Sample Prep Kit protocol with an input amount of 50 ng DNA.

For nanopore sequencing, libraries were prepared using the Ligation Sequencing Kit 2D (Oxford Nanopore Technologies Ltd., Oxford, UK) according to the manufacture protocol with DNA inputs ranging between 715 and 1500 ng.

To analyze the metatranscriptomes, RNA was prepared using the ScriptSeq Complete Kit for Bacteria according to its low input protocol (Illumina, USA). Depending on the available amount of starting material, 20 ng or 100 ng of total RNA per sample was used.

Prior to sequencing, all libraries were quality controlled using the HS NGS Fragment Analysis Kit on a Fragment Analyzer (AATI, USA).

### Nucleic acid sequencing

Libraries for the assembly as well as for RNA seq were sequenced by a HiSeq 2500 (Illumina, USA) for 150 cycles in paired-end mode with an average sequencing depth of approximately 38 million reads. Same settings were chosen for the additional samples from biogas plant 5–7, with expected sequence depth of only approximately 15 million reads per sample. Libraries from R1 and R2 were also sequenced by a HiSeq 2500 (Illumina, USA) with a 150 bp paired-end reads and average sequencing depth of approximately 13 million read pairs per sample. To analyze the metatranscriptomes for R1 and R2, Illumina sequencing was performed applying 140 cycles in single-end mode with an average depth of 34 million reads per sample.

Each library prepared for long-read sequencing was loaded on Spoton Flow Cells MkI R9.4 and sequenced on a MinION according to the manufacture’s instructions for 48 h using MinKnow v.1.1.21.1 software, followed by a base calling on Metrichor cloud-based service (Oxford Nanopore Technologies Ltd., Oxford, UK).

### Bioinformatics workflow—Illumina-generated sequences

All samples were de-multiplexed using Illumina’s bcl2fastq (v1.84) software with default settings for adapter trimming (at least 90% of bases should match) and allowing no mismatch per sequencing bar code (–mismatches 0). Raw Illumina reads were cleared from potential adapter contamination, quality controlled, and, if necessary, trimmed in paired-end mode using BBDuk from the BBMap package version 34.41 (https://sourceforge.net/projects/bbmap/). To pass the quality filter, read quality needed to surpass a Phred score of 20 and achieve a minimal length of 50 bp after trimming of low quality and adapter bases.

Additional data quality control measures were taken: each sample was tested before and after trimming with the FastQC to evaluate per base sequence quality, average base composition, GC content, sequence length distribution and adapter contaminations (http://www.bioinformatics.babraham.ac.uk/projects/fastqc/).

### Oxford Nanopore Technology (ONT)-generated sequences

Raw reads (FAST5) were acquired using MinKnow v.1.1.21.1 software (samples neo19, neo20, neo21, neo23) [28, 29]. Base calling for MinION data is performed using a cloud-based service provided by ONT—Metrichor (metrichor.com). Metrichor applies HMM-based methods to extract sequence information from raw data. Active internet connection is required to upload raw reads to the Metrichor and download resulting fastq files. Base-called ONT reads were cleared from potential adapter contamination, quality controlled, and trimmed in single-end mode using BBDuk from the BBMap package version 34.41 (https://sourceforge.net/projects/bbmap/). To pass the quality filter, average read quality needed to surpass a Phred score of 7 and achieve a minimal length of 50 bp after trimming of low quality and adapter bases.

The quality of resulting sequences was controlled with Poretools [30] and NanoPlot [31] packages.

### Hybrid metagenome assembly

The assembly was executed on a computational cluster running under CentOS 6.9 by SPAdes v.3.10.1 with merged ONT dataset and four paired-end Illumina datasets [32]. Since SPAdes v.3.10.1 could not be applied to metagenomic samples generated by different sequencing technologies, we treated metagenomics sample as if they were from a single organism. SPAdes was executed with automatic coverage cutoff (–cov-cutoff auto), mismatch correction enabled (–mismatch-correction) and iterative k-mer lengths optimization (-k 21, 33, 55, 77, 127). All contigs smaller than 1000 bp were considered as singletons and discarded as they were not supported by other reads.

The quality of the draft metagenome was further improved with the NanoPolish v.0.6.1 software package, which can utilize information from raw MinION reads [33], followed by two iterations of Pilon v.1.22 [34]. Pilon, in contrast to NanoPolish, uses paired-end Illumina short reads for corrections. Nano-polishing was conducted according to a standard procedure described in the documentation to the software package: initial draft assembly is indexed and mapped to MinION reads converted into a single fasta file, using the BWA-MEM algorithm [35, 36]. The output BAM file is indexed and sorted with the Samtools [37, 38] and then used as input by NanoPolish together with the metagenome draft assembly and MinION reads.

The improved sequence in turn is used as an input to Pilon to correct any inconsistency between draft assembly and evidences in the collection of paired-end Illumina reads. Pilon requires mapping of all Illumina reads to a draft assembly at the first step, followed by sorting and indexing of mapped reads. We used NextGenMap v.0.4.12 in paired-end mode for mapping [39] and Samtools for indexing and sorting of mapped reads. For subsequent iteration, Pilon results of the previous iteration were used as an input.

### Binning

For a binning procedure of acquired assembly, we have applied the MetaWatt package specialized for metagenomic samples [40]. MetaWatt utilizes multivariate statistics of tetranucleotide frequencies combined with interpolated Markov models. The MetaWatt pipeline includes parallel ORF prediction with Prodigal [41], taxonomical classification and blastx search with sequence aligner DIAMOND [42], predicting tRNAs with Aragorn [43], taxonomical classification based on conserved genes with hmmsearch [44] as well as usearch algorithms [45].

Only bins with profile completeness of 10% or more (based on mapping to a set of conservative genes) and with coverage of at least 50% or more (mapping of at least 2 out of 4 metagenomic Illumina samples) were selected for final assembly.

Selected bins were classified to taxa by the “DIAMOND blastx” included in the MetaWatt workflow [42]. Each contig belonging to a particular bin was first split into 1000 bp fragments, and then the software module performed a blastx search for each fragment against the RefSeq database, containing only organisms with complete reference genomes. For taxonomical classification of the bin, the total number of top-scoring blastx hits is counted for every fragment of every contig. The sum of all counts for each taxon divided by the number of fragments in the bin was calculated. Bins were assigned to taxonomic level according to closest common ancestor of top-scoring taxons.

### Functional annotation of metagenome hybrid assembly

The final hybrid metagenome assembly was annotated using Prokka v.1.11—a tool for rapid prokaryotic genome annotation [46]. Prokka was executed for Archaea, Bacteria and Viruses kingdoms separately enabling improved gene prediction for highly fragmented genomes with the –metagenome flag. Final annotation includes annotations of individual bins according to their taxonomical classifications assigned by MetaWatt. Annotation for bins sharing mixed taxonomic background was produced by merging corresponding Bacteria and Archaea annotations. This was accomplished by the custom Perl script “merge_gff_annotation.pl”, which complemented predicted CDS from bacteria with predicted and annotated CDS from archaea. We consider predicted CDS to be “annotated” when either gene ID or EC number is known.

To wrap up the functional annotation generated by Prokka, we additionally annotated every genomic bin with InterProScan v5.30 [47]. InterProScan was executed with all default databases, default analysis algorithms selection, additional pathways lookup algorithm and DNA sequence as a search template. Resulting tab-delimited TSV text files generated for every genomic bin were either merged together to generate a full annotation or used separately for follow-up analysis.

### Anaerobic digestion pathway map

The comprehensive pathway overview map, summarizing and reconstructing all biological processes relevant to anaerobic digestion pathways of carbohydrates lipids and proteins, was created by detailed literature and database research (KEGG, MetaCyc, BRENDA). A corresponding list of references is provided separately as Additional file 8.

### Biogas production specific EC reference sequence collection (ERSC)

The EC number (enzyme commission) is a numerical classification scheme for enzymes and enzyme-catalyzed reactions derived from different organisms. We downloaded all reference sequences from KEGG or NCBI databases belonging to enzymes relevant for anaerobic digestion. All downloaded sequences were supplemented using BioMETHA sequences specific to the previously described enzymatic map (Additional file 7). EC sequences are assigned to metabolic modules; whereas, the term metabolic module refers to the complement of enzymes involved in the conversion of a given substrate and a metabolic process, respectively.

## Additional files

**Additional file 1.** Summary of metagenomic (DNA) and metatranscriptomic (RNA) sequenced samples.

**Additional file 2.** Summary of genomic bins including estimated profile completeness, number of deduced coding sequences and EC numbers.

**Additional file 3.** Representation of GO terms from top 500 expressed transcripts.

**Additional file 4.** List of GO terms from top 500 expressed transcripts.

**Additional file 5.** Heatmap of top 500 differentially expressed transcripts.

**Additional file 6.** Annotated list of top 500 differentially expressed transcripts.

**Additional file 7.** Pathway map of EC numbers involved in anaerobic digestion and methanogenesis.

**Additional file 8.** Reference list used to generate pathway map.

**Additional file 9.** Summary of EC numbers with assignments to metabolic modules.

**Additional file 10.** Heatmap of log2-transformed RPKM values of enzymes associated with methanogenesis.

## Abbreviations

AD: anaerobic digestion
BioMETHA: Biogas Metagenomics Hybrid Assembly
CBT: continuous biogas test
EC: enzyme commission
ERSC: EC reference sequence collection
MiMaS: microbial management system
NGS: next-generation sequencing
ONT: Oxford Nanopore Technology
WGS: whole genome shotgun
Bgp: biogas plant
