# Supplementary material for: Genetic repertoires of anaerobic microbiomes driving generation of biogas
Source: Biotechnol Biofuels. 2018 Sep 20;11:255. doi: 10.1186/s13068-018-1258-x (PMC6146632; doi:10.1186/s13068-018-1258-x)
Supplement: Supplementary file 1 — Additional file 1. Summary of metagenomic (DNA) and metatranscriptomic (RNA) sequenced samples. [file 13068_2018_1258_MOESM1_ESM.docx]

# Additional file 1

**
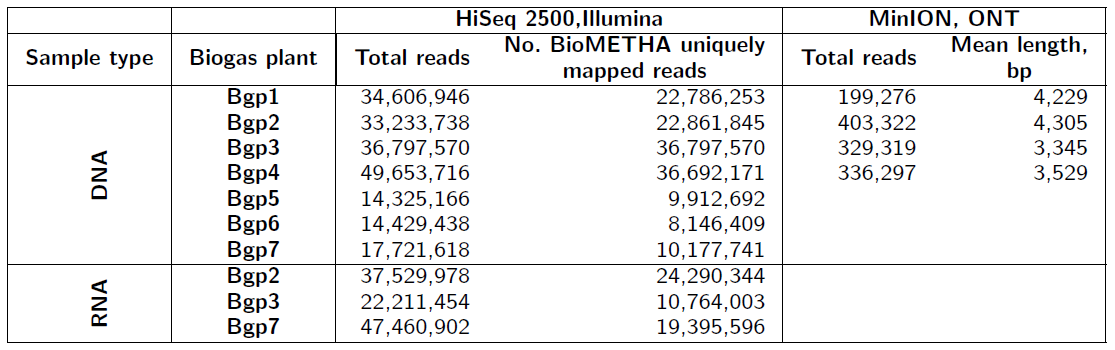
**

**Samples overview. Metagenomic and Metatranscriptomic samples sequencing statistics**
