# Supplementary material for: Genetic repertoires of anaerobic microbiomes driving generation of biogas
Source: Biotechnol Biofuels. 2018 Sep 20;11:255. doi: 10.1186/s13068-018-1258-x (PMC6146632; doi:10.1186/s13068-018-1258-x)
Supplement: Supplementary file 3 — Additional file 3. Representation of GO terms from top 500 expressed transcripts. [file 13068_2018_1258_MOESM3_ESM.docx]

# Additional file 3


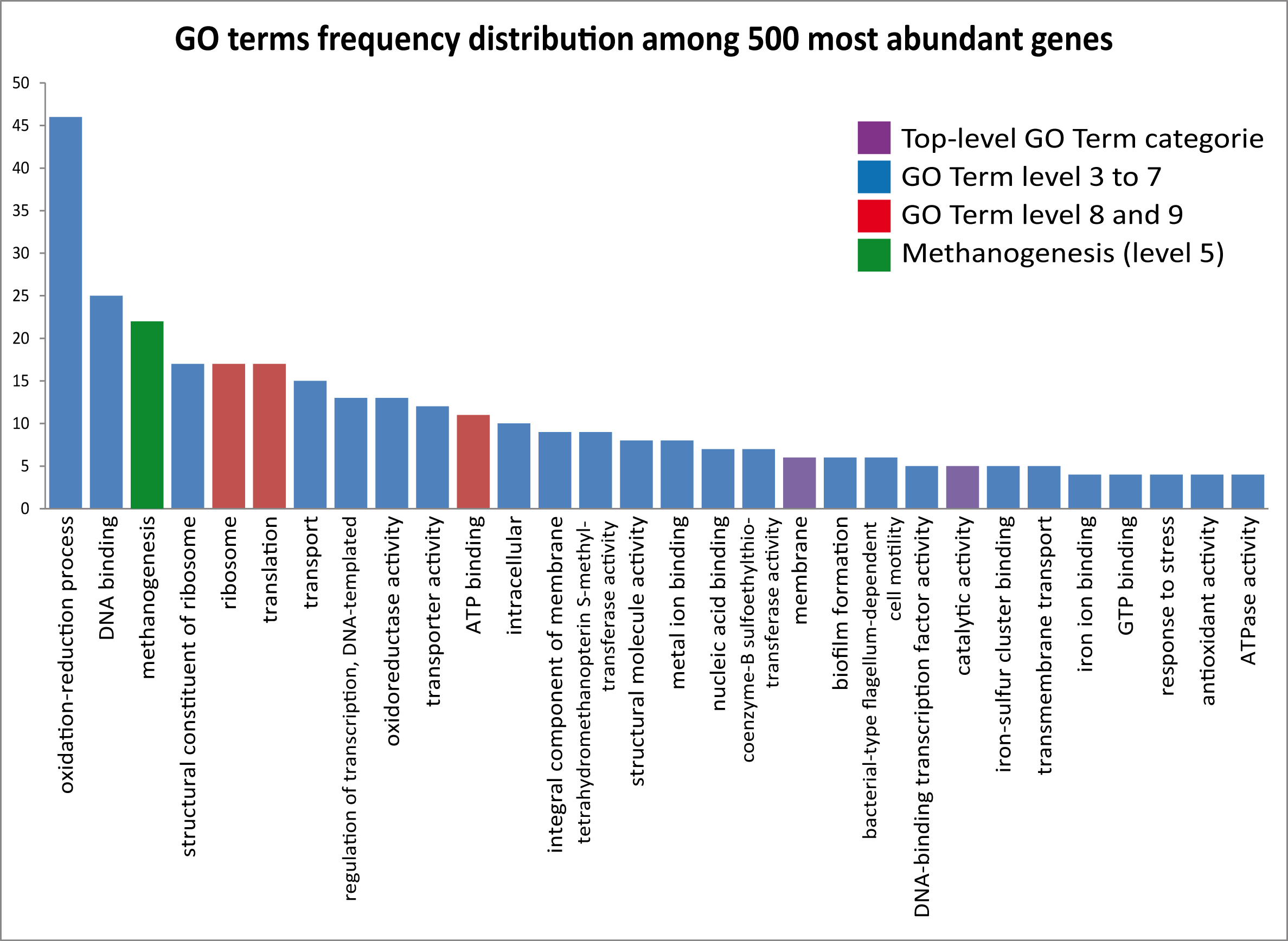


**GO terms frequency distribution.**

The frequency distribution of GO terms among most abundant genes in methane producing laboratory reactors (R1, R2, time 42, and 48 hours), based on mean log2 RPKM calculations.
