# Supplementary material for: Genetic repertoires of anaerobic microbiomes driving generation of biogas
Source: Biotechnol Biofuels. 2018 Sep 20;11:255. doi: 10.1186/s13068-018-1258-x (PMC6146632; doi:10.1186/s13068-018-1258-x)
Supplement: Supplementary file 5 — Additional file 5. Heatmap of top 500 differentially expressed transcripts. [file 13068_2018_1258_MOESM5_ESM.docx]

# Additional file 5


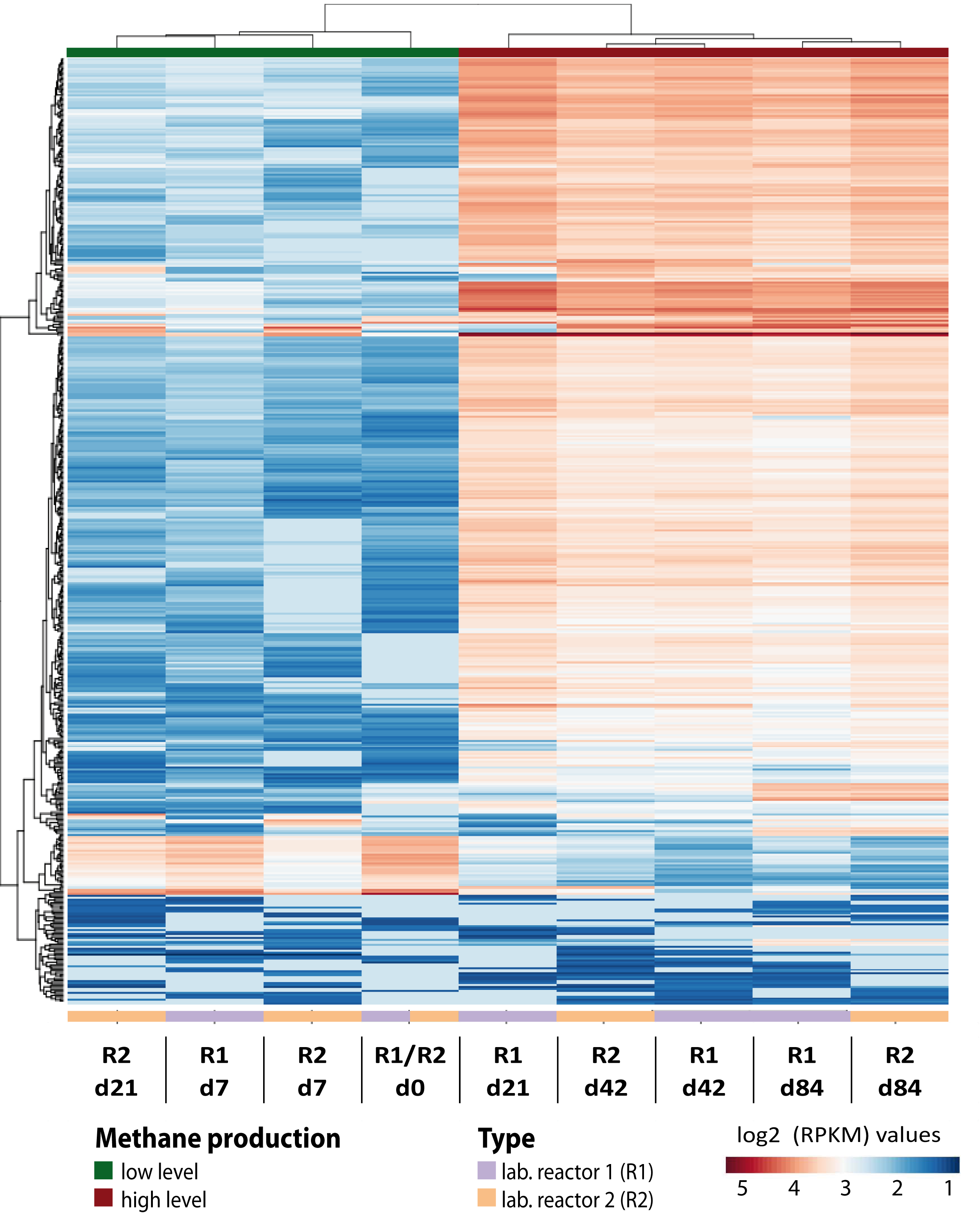


**Comparative metatranscriptomic analysis of laboratory reactors.**

Heatmap colors representing gene log2 RPM values. Hierarchical clustering was done for samples as well as for genes. Corresponding dendrogram on the top shows clustering of biogas plants and laboratory reactors. The dendrogram on the left demonstrating clustering of genes.
