# Supplementary figures and images for: Genetic repertoires of anaerobic microbiomes driving generation of biogas
Source: Biotechnol Biofuels. 2018 Sep 20;11:255. doi: 10.1186/s13068-018-1258-x (PMC6146632; doi:10.1186/s13068-018-1258-x)

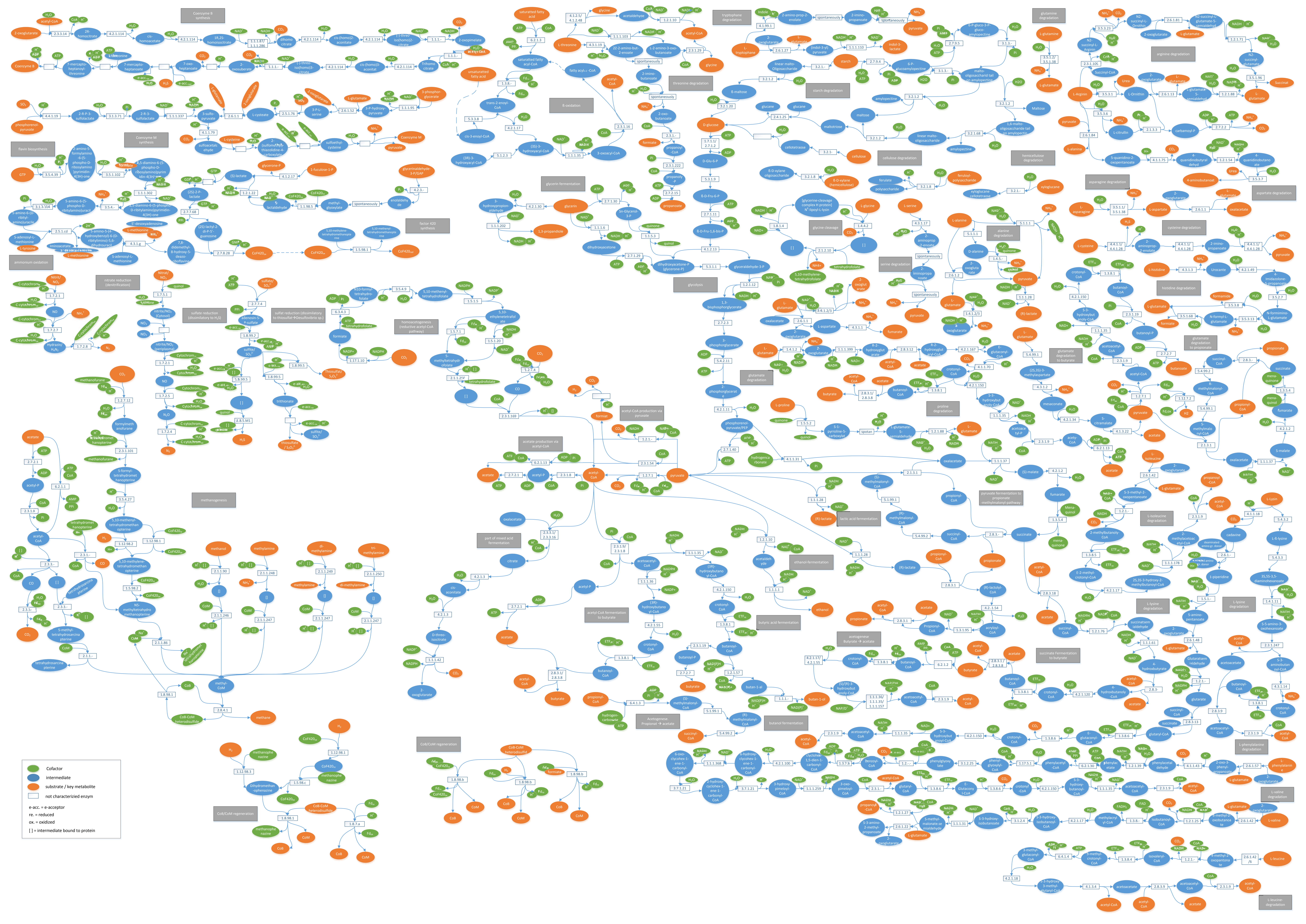

Supplement: Supplementary file 7 — Additional file 7. Pathway map of EC numbers involved in anaerobic digestion and methanogenesis. [file 13068_2018_1258_MOESM7_ESM.pdf]
