# Supplementary material for: Genetic repertoires of anaerobic microbiomes driving generation of biogas
Source: Biotechnol Biofuels. 2018 Sep 20;11:255. doi: 10.1186/s13068-018-1258-x (PMC6146632; doi:10.1186/s13068-018-1258-x)
Supplement: Supplementary file 8 — Additional file 8. Reference list used to generate pathway map. [file 13068_2018_1258_MOESM8_ESM.docx]

**Additional file 8**

**References for EC pathway map**

1. Albert, G.M., J.W. Foster, and M.P. Spector. *Microbial physiology*. New York: Wiley-Liss. Inc., 2002.
2. Allen, S.H.G., et al., *Purification and properties of enzymes involved in the propionic acid fermentation*. Journal of Bacteriology, 1964. **81**(1): p. 171-187.
3. Bapteste, E., C. Brochier, and Y. Boucher. *Higher-level classification of the archaea: evolution of methanogenesis and methanogens*. Archaea, 2005. **1**(5): p. 353-363.
4. Barbirato, F., et al., *Glycerol fermentation by a new 1,3-propanediol-producing microorganism: Enterobacter agglomerans*. Appl. Microbiol. Biotechnol., 1995. **43**(5): p. 786-793.
5. Barker, H.A., *Amino acid degradation by anaerobic bacteria*. Ann. Rev. Biochem., 1981. **50**: p. 23-40.
6. Blaut, M., *Metabolism of methanogens*. Antonie van Leeuwenhoek, 1994. **66**(1-3): p. 187-208.
7. Bryant, M. P., *Microbial methane production - theoretical aspects*. Journal of Animal Science, 1979. **48**(1): p. 193-201.
8. Choudhary, M.K., et al., *Re-examination of metabolic fluxes in Escherichia coli during anaerobic fermentation of glucose using ^13^C labeling Experiments and 2-dimensional Nuclear Magnetic Resonance (NMR) spectroscopy*. Biotechnology and Bioprocess Engineering, 2011. **16**(3): p. 419-437.
9. Converti, A., and P. Perego. *Use of carbon and energy balances in the study of the anaerobic metabolism of Enterbacter aerogenes at variable starting glucose concentrations*. Appl. Microbial. Biotechnol., 2002. **59**(2-3): 303-309.
10. Batstone,D.J., *High rate anaerobic treatment of complex wastewater*. The University of Queensland, 2000.
11. Denman, S.E., et al., *Metagenomic analysis of the rumen microbial community following inhibition of methane formation by a halogenated methane analog*. Fontiers in Microbiology, 2015. **6**: p. 1-12.
12. Deppenmeier, U., V. Müller and G. Gottschalk. *Pathways of energy conservation in methanogenic archaea*. Arch Microbiol, 1996. **165**(3): p. 149-163.
13. Diekert, G., and G. Wohlfarth. *Metabolism of homoacetogens*. Antonie van Leeunwenhoek, 1994. **66**(1-3): p. 209-221.
14. Drake, H.L., A.S. Gößner, and S.L. Daniel. *Old Acetogens, New Light*. Ann. N. Y. Acad. Sci., 2008. **1125**(1): p. 100-128.
15. Eden, G., and G. Fuchs. *Total sythesis of acetyl coenzyme A involved in autotrophic CO_2_ fixation in Acetobacterium woodii*. Arch Microbiol, 1982. **133**(1): p. 66-74.
16. Gavala, H.N., I. Angelidaki, and B.K. Ahring. *Kinetics and modeling of anaerobic digestion process.* In Biomethanation I, by B.K. Ahring. Heilderberg Berlin: Springer, 2003.
17. Gonzàlez-Cabaleiro, R., J.M. Lema, and J. Rodriguez. *Metabolic energy-based modelling explains product yielding in anaerobic mixed culture fermentations*. PLOS ONE, 2015. **10**(5): p 1-17.
18. Harper, S.R., and F.G. Pohland. *Recent developments in hydrogen management during anaerobic biological wastwater treatment*. Biotechnology and Bioengineering, 1986. **28**(4): p. 585-602.
19. Hetzel, M., et al., *Acryloyl-CoA reductase from Clostridium propionicum*. Eur. J. Biochem, 2003. **270**(5): p. 902-910.
20. Ivan A., Berg. *Ecological aspects of the distribution of different autotrophic CO_2_ fixation pathways*. Applied and Environmental Microbiology, 2011. **77**(6): p. 1925-1936.
21. Kleerebezem, R., and A.J.M. Stams. *Kinetics of syntrophic cultures: A theoretical treatise on butyrate fermentation*. Biotechnology and Bioengineering, 2000. **67**(5): p. 529-543.
22. Kosaka, T., et al., *Reconstruction and regulation of the central catabolic pathway in the thermophilic propionate-oxidizing syntroph Pelotomaculum thermopropionicum*. Journal of Bacteriology, 2006. **188**(1): p. 202-210.
23. Lee, H.-S., M. B. Salero, and B. E. Rittmann. *Thermodynamic evaluation on H_2_ production in glucose fermentation*. Environ. Sci. Technol., 2008. **42**(7): p. 2401-2407.
24. Liu, Y., and W. Whitman. *Metabolic, phylogenetic and ecological diversity of the methanogenic archaea*. Ann. N.Y. Acard. Sci., 2008. **1125**: p. 171-189.
25. Macfarlane, S., and G.T. Macfarlane. *Regulation of short-chain fatty acid production*. Proceedings of the Nutrition Society, 2003. **62**(1): p. 67-72.
26. Macy, J.M., L.G. Ljungdahl, and G. Gottschalk. P*athway of succinat and propionate formation in Bacteroides fragilis*. Journal of Bacteriology, 1978. **134**(1): p. 84-91.
27. Moscoviz, R., E. Trably, and N. Bernet. *Consistent 1,3-propanediol production from glycerol in mixed culture fermentation over a wide rang of pH*. Biotechnol Biofuels, 2016. **9**(32): p. 1-11.
28. Müller, N., et al., *Syntrophic butyrat and propionate oxidation processes: from genomes to reaction mechanisms*. Environmental Microbiology Reports, 2010. **2**(4): p. 489-499.
29. Müller, V., *Energy Conversion in acetogenic bacteria*. Applied and Environmental Microbiology, 2003. **69**(11): p. 6345-6353.
30. Müller, V., *Bacterial fermentation*. Encyclopedia of Life Sciences, 2001: p. 1-7.
31. Nagase, M., and T. Matsuo. *Interaction between amino-acid-degrading bacteria and methanogenic bacteria in anerobic digestion*. Biotechnology and Bioengineering, 1982. **24**(10): p. 2227-2239.
32. Paulo da Silva, G., M. Mack, and J. Contiero. *Glycerol: A promising and abundant carbon source for industrial microbiology*. Biotechnology Advances, 2009. **27**(1): p. 30-39.
33. Pryde, S.E., et al., *The microbiology of butyrate formation in the human colon*. FEMS Microbiology Letters, 2002. **217**(2): p. 133-139.
34. Ramsay, I.R., and P.C. Pullammanappallil. *Protein degradation during abaerobic wastewater treatment: derivation of stoichiometry*. Biodegradation, 2001. **12**(4): p. 247-257.
35. Sawers, G., *The anaerobic degradation of L-serine and L-threonine in enterobacteria: networks of pathways and regulatory signals*. Arch Microbiol, 1998. **171**(1): p. 1-5.
36. Schink, B., *Energetics of syntrophic cooperation in methanogenic degradation*. Microbiology and Molecular Biology Reviews, 1997. **61**(2): p. 262-280.
37. Sciel-Bengelsdorf, B., and P. Dürre. P*athway engineerind and synthetic biology using acetogens*. FEBS Letters, 2012. **586**(15): p. 2191-2198.
38. Seidhar, J., and M.A. Eiteman. *Metabolic flux analysis of Clostridium thermosuccinogenes*. Applied Biochemistry and Biotechnology, 2001. **94**(1): p. 51-69.
39. Sousa, D.Z., et al., *Ecophysiology of syntrophic communities that degrade saturated and unsaturated long-chain fatty acids*. FEMS Microbiol Ecol, 2009. **68**(3): p. 257-272.
40. Stams, A.J.M., *Metabolic interactions between anaerobic bacteria in methanogenic environments*. Antonie van Leeuwenhoek, 1994. **66**(1-3): p. 271-294.
41. Stams, A.J.M., and C.M. Plugge. *Electron transfer in syntrophic communities of anaerobic bacteria and archaea*. Nature Reviews Microbiology, 2009. **7**(8): p. 568-577.
42. Stams, A.J.M., S.J.W.H. Oude Elferink, and P. Westermann. *Metabolic interaction between methanogenic consortia and anaerobic respiring bacteria*. In Biomethanation I, by B.K. Ahring, 31-56. Berlin Heidelberg: Springer, 2003.
43. Thauer, R.K., et al., *Methanogenic archaea: ecologically relevant differences in energy conversion*. Nature Reviews Microbiology, 2008. **6**: p. 579-591.
44. Thauer, R.K., D. Möller-Zinkhan, and A.M. Spormann. B*iochemistry of acetate catabolism in anerobic chemotrophic bacteria*. Ann. Rev. Microbiol., 1989. **43**: p. 43-67.
45. Thauer, R.K., K. Jungermann, and K. Decker. E*nergy conversion in chemotrophic anaerobic bacteria*. Bacteriological Reviews, 1977. **41**(1): p. 100-180.
46. W. Buckel, R. K. Thauer. *Energy conversion via electron bifurcating ferredoxin reduction and proton/Na+ translocating ferredoxin oxidation*. Biochimica et Biophysic Acta, 2013. **1827**(2): p. 94-113.
47. Weiss, D.S. and R.K. Thauer. *Methanogenesis and the unity of biochemistry*. Cell, 1993. **72**: 819-822.
48. Zeikus, J.G., *Chemical and fuel production by anaerobic bacteria*. Ann. Rev. Microbiol., 1980. **34**: p. 423-464.

49. Caspi, R., et al., *The MetyCyc database of metabolic pathways and enzymes and the BioCyc collection of pathway/genome databases*. Nucleic Acids Res., 2012. **40**: p. D742-753.
