# Supplementary material for: Genetic repertoires of anaerobic microbiomes driving generation of biogas
Source: Biotechnol Biofuels. 2018 Sep 20;11:255. doi: 10.1186/s13068-018-1258-x (PMC6146632; doi:10.1186/s13068-018-1258-x)
Supplement: Supplementary file 10 — Additional file 10. Heatmap of log2-transformed RPKM values of enzymes associated with methanogenesis. [file 13068_2018_1258_MOESM10_ESM.docx]

# Additional file 10


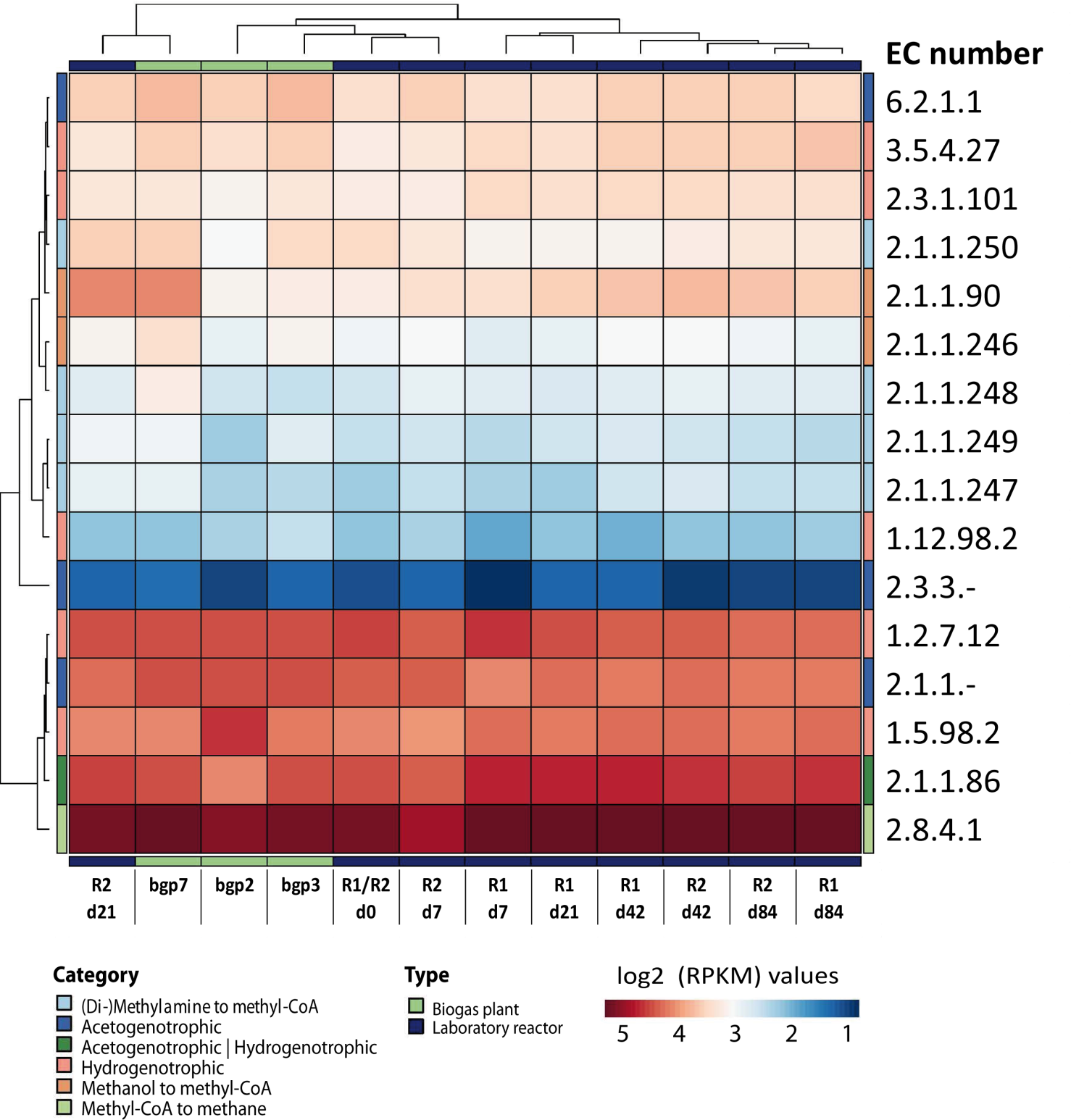


**Comparative metatranscriptomic analysis of methanogenesis metabolic module.**

Heatmap of log2-transformed RPKM values of enzymes associated with methanogenesis metabolic module.
